# Supplementary material for: Convergence of immune escape strategies highlights plasticity of SARS-CoV-2 spike
Source: PLoS Pathog. 2023 May 1;19(5):e1011308. doi: 10.1371/journal.ppat.1011308 (PMC10174534; doi:10.1371/journal.ppat.1011308)
Supplement: S1 Table — (DOCX) [file ppat.1011308.s001.docx]

**S1 Table. Overview of collected sequences with large deletions in the spike variants from the COV3001 trial**

| Sequence ID | Collection date | Country code | Lineage | #Deletions | #Mutations | P9L | S46L | **T63del** | **Del 64-75** | **T76del** | **Del 136-144** | **R246del** | R246N | **Del 247-252** | D253N | **D253del** | **Del 258-264** | D287N | L452Q | E471Q | E484K | F490S | S494P | D614G | N703S | T859N | V1176F |
| --- | --- | --- | --- | --- | --- | --- | --- | --- | --- | --- | --- | --- | --- | --- | --- | --- | --- | --- | --- | --- | --- | --- | --- | --- | --- | --- | --- |
| 1 | 12-Jan-21 | BR | B.1.1.294 | 16 | 21 | x |  |  |  |  | ● |  |  |  |  |  | ● |  |  |  | x |  | x | x |  |  | x |
| 2 | 13-Jan-21 | PE | C.37 | 20 | 26 |  |  |  | ● | ● |  |  | x | ● |  | ● |  |  | x | x |  | x |  | x |  | x |  |
| 3 | 17-Jan-21 | BR | B.1.1.294 | 16 | 21 | x |  |  |  |  | ● |  |  |  |  |  | ● |  |  |  | x |  | x | x |  |  | x |
| 4 | 9-Feb-21 | PE | C.37 | 20 | 25 |  |  |  | ● | ● |  |  | x | ● |  | ● |  |  | x |  |  | x |  | x |  | x |  |
| 5 | 23-Feb-21 | PE | C.37 | 20 | 26 |  |  | ● | ● |  |  | ● |  | ● | x |  |  |  | x | x |  | x |  | x |  | x |  |
| 6 | 24-Feb-21 | PE | C.37 | 20 | 26 |  |  | ● | ● |  |  | ● |  | ● | x |  |  |  | x | x |  | x |  | x |  | x |  |
| 7 | 16-Mar-21 | PE | C.37 | 20 | 25 |  |  | ● | ● |  |  | ● |  | ● | x |  |  |  | x |  |  | x |  | x |  | x |  |
| 8 | 2-Apr-21 | PE | C.37 | 20 | 27 |  | x | ● | ● |  |  | ● |  | ● | x |  |  |  | x |  |  | x |  | x | x | x |  |
| 9 | 5-Apr-21 | AR | C.37 | 20 | 26 |  |  | ● | ● |  |  | ● |  | ● | x |  |  | x | x |  |  | x |  | x |  | x |  |
| 10 | 15-Apr-21 | PE | C.37 | 20 | 25 |  |  | ● | ● |  |  | ● |  | ● | x |  |  |  | x |  |  | x |  | x |  | x |  |
| 11 | 26-May-21 | PE | C.37 | 20 | 25 |  |  | ● | ● |  |  | ● |  | ● | x |  |  |  | x |  |  | x |  | x |  | x |  |
| 12 | 25-Jun-21 | AR | C.37 | 20 | 25 |  |  | ● | ● |  |  | ● |  | ● | x |  |  |  | x |  |  | x |  | x |  | x |  |
