# Supplementary material for: Convergence of immune escape strategies highlights plasticity of SARS-CoV-2 spike
Source: PLoS Pathog. 2023 May 1;19(5):e1011308. doi: 10.1371/journal.ppat.1011308 (PMC10174534; doi:10.1371/journal.ppat.1011308)
Supplement: S2 Table — (DOCX) [file ppat.1011308.s002.docx]

**S2 Table. Sera from COVID-19 patients**

| **Name** | **BioIVT Lot** | **Severity of symptoms** |
| --- | --- | --- |
| E012625-1 | HMN464287 | Mild |
| E012625-2 | HMN464288 | Mild |
| E012625-3 | HMN374190-SR75 | Asymptomatic |
| E012625-4 | HMN374191-SR70 | Critical |
| E012625-5 | HMN374192-SR85 | Mild |
| E012625-6 | HMN374193 -SR95 | Asymptomatic |
| E012625-7 | HMN374194-SR80 | Mild |
| E012625-8 | HMN374195-SR85 | Asymptomatic |
| E012625-9 | HMN374196-SR80 | Asymptomatic |
| E012625-10 | HMN374197-SR85 | Moderate |
