# Supplementary material for: Convergence of immune escape strategies highlights plasticity of SARS-CoV-2 spike
Source: PLoS Pathog. 2023 May 1;19(5):e1011308. doi: 10.1371/journal.ppat.1011308 (PMC10174534; doi:10.1371/journal.ppat.1011308)
Supplement: S3 Table — (DOCX) [file ppat.1011308.s003.docx]

**S3 Table. Data collection, reconstruction, and model refinement statistics**

|  | ΔN135 (C1)  EMD-29455, PDB: 8FU8 | ΔN135 (C3)  EMD-29454, PDB: 8FU7 | ΔN25 (C1)  EMD-29456, PDB: 8FU9 |
| --- | --- | --- | --- |
| **Data collection** |  |  |  |
| Microscope | Titan Krios | | Titan Krios |
| Voltage (keV) | 300 | | 300 |
| Nominal magnification | 105000 x | | 105000 x |
| Exposure navigation | Image Shift | | Image Shift |
| Electron exposure (e /Å^2^) | 51.98 | | 52.25 |
| Total exposure time (sec) | 1.4 | | 1.4 |
| Detector | K3 Summit | | K3 Summit |
| Pixel size (Å)* | 0.832 | | 0.832 |
| Defocus range (µm) | -1.2 to -1.8 | | -1.2 to -1.6 |
| Micrographs Used | 20,732 | | 18,021 |
| Final Refined particles (no.) | 378,064 | 115,817 | 207,525 |
|  |  |  |  |
| **Reconstruction** |  |  |  |
| Symmetry imposed | C1 | C3 | C1 |
| Resolution (global) |  |  |  |
| FSC 0.143 | 3.08 Å | 3.21 Å | 3.52 Å |
| Applied B-factor (Å^2^) | -101 | -118 | -125 |
|  |  |  |  |
| **Refinement** |  |  |  |
| Protein residues | 3137 | 3108 | 3147 |
| Map Correlation Coefficient (Main chain) | 0.8439 | 0.8216 | 0.7131 |
| R.m.s deviations |  |  |  |
| Bond lengths (Å) | 0.012 | 0.012 | 0.011 |
| Bond angles (°) | 1.08 | 1.08 | 1.09 |
| Ramachandran |  |  |  |
| Outliers | 1.05 % | 0.87 % | 0.76 % |
| Allowed | 5.36 % | 5.18 % | 5.63 % |
| Favored | 93.59 % | 93.95 % | 93.61 % |
| Rotamer outliers | 0.07 % | 0.11 % | 0.07 % |
| MolProbity score | 1.36 | 1.35 | 1.36 |
| EMRinger score | 3.35 | 2.82 | 1.94 |
| Clashscore (all atoms) | 1.77 | 1.84 | 1.77 |

*Calibrated pixel size at the detector
