# Supplementary material for: Convergence of immune escape strategies highlights plasticity of SARS-CoV-2 spike
Source: PLoS Pathog. 2023 May 1;19(5):e1011308. doi: 10.1371/journal.ppat.1011308 (PMC10174534; doi:10.1371/journal.ppat.1011308)
Supplement: S4 Table — (DOCX) [file ppat.1011308.s004.docx]

**S4 Table. Mutations leading to DS_15-136_ loss in lineages**

| **Mutation SP** | **Mutation 136** | **Count** | **Lineage** | **Percent of mutation within lineage** | | | |
| --- | --- | --- | --- | --- | --- | --- | --- |
| S13I | WT | 45886 | B.1.429 | 89.30% | | |  |
| S13I | WT | 19863 | B.1.427 | 80.81% | | |  |
| S13I | WT | 1764 | B.1.427 | 7.18% | | |  |
| S13I | WT | 1375 | B.1.429 | 2.68% | | |  |
| L5F+S13I | WT | 784 | B.1.429 | 1.53% | | |  |
| P9L | C136del | 690 | B.1.640.1 | 60.79% | | |  |
| L5F+S13I | WT | 579 | B.1.427 | 2.36% | | |  |
| P9L | WT | 416 | B.1.640.1 | 36.65% | | |  |
| P9L+S13I | WT | 382 | B.1.427 | 1.55% | | |  |
| P9L | C136F | 300 | C.1.2 | 83.57% | | |  |
| P9L | C136F | 187 | B.1.630 | 92.57% | | |  |
| P9L | C136del | 175 | AT.1 | 88.38% | | |  |
| P9L | WT | 171 | P.3 | 27.23% | | |  |
| P9L | WT | 116 | C.38 | 84.67% | | |  |
| S13I | WT | 49 | B.1.429.1 | 100.00% | | |  |
| S13I | WT | 47 | A.2.5 | 1.90% | | |  |
| P9L | WT | 35 | N.10 | 100.00% | | |  |
| P9L | WT | 30 | B.1.640.2 | 46.88% | | |  |
| WT | C136F | 30 | C.1.2 | 8.36% | | |  |
| P9L | WT | 29 | C.1.2 | 8.08% | | |  |
| P9L | C136del | 27 | B.1.640.2 | 42.19% | | |  |
| P9L | WT | 27 | BA.2.3.21 | 75.00% | | |  |
| S12F+S13I | WT | 26 | C.36.3 | 1.28% | | |  |
| P9L | C136F | 24 | B.1.1.524 | 54.55% | | |  |
| P9L | WT | 23 | C.39 | 100.00% | | |  |
| P9L | WT | 20 | AT.1 | 10.10% | | |  |
| P9L | WT | 20 | B.1.1.524 | 45.45% | | |  |
| S13I | WT | 20 | B.1.108 | 9.48% | | |  |
| Q14del+C15del | WT | 14 | A.2.5.2 | 1.22% | | |  |
| P9L | WT | 13 | P.3 | 2.07% | | |  |
| P9L | WT | 11 | CP.1 | 1.57% | | |  |
| P9L | WT | 10 | B.1.630 | 4.95% | | |  |
| P9L | C136F | 10 | C.1 | 1.94% | | |  |
| L5F+P9L | WT | 9 | BA.1.6 | 1.22% | | |  |
| P9L | WT | 8 | B.1.149 | 2.53% | | |  |
| L5F+Q14del+C15del | C136Y | 7 | B.1.638 | 63.64% | | |  |
| L5F+P9L | C136del | 7 | B.1.640.2 | 10.94% | | |  |
| P9L | WT | 6 | BT.2 | 1.17% | | |  |
| P9L | WT | 5 | B.1.1.273 | 3.25% | | |  |
| WT | C136F | 5 | B.1.630 | 2.48% | | |  |
| P9L | WT | 4 | B.1.149 | 1.27% | | |  |
| P9L | WT | 3 | C.23 | 1.48% | | |  |
| Q14del+C15del | WT | 3 | W.1 | | 2.65% |  |  |
