# Supplementary material for: Convergence of immune escape strategies highlights plasticity of SARS-CoV-2 spike
Source: PLoS Pathog. 2023 May 1;19(5):e1011308. doi: 10.1371/journal.ppat.1011308 (PMC10174534; doi:10.1371/journal.ppat.1011308)
Supplement: S5 Table — (DOCX) [file ppat.1011308.s005.docx]

**S5 Table. Local occurrences of DS_15-136_ loss in the Delta and Omicron lineages.**

| **Lineage** | **C15*** | **C136** | **both** | **Country** | **Period** | **#Sequences** | **Lineage percentage** |
| --- | --- | --- | --- | --- | --- | --- | --- |
| BA.2.3.21 | × |  |  | Philippines | 2022-Q2 | 6 | 100.00% |
| BA.2.3.21 | × |  |  | Philippines | 2022-Q3 | 6 | 100.00% |
| BA.2.3.21 | × |  |  | Philippines | 2022-Q4 | 6 | 100.00% |
| BA.1.6 | × |  |  | Denmark | 2022-Q1 | 9 | 39.13% |
| BA.1.1.14 | × |  | × | Slovenia | 2022-Q1 | 11 | 28.21% |
| BA.1.15 | × | × |  | USA | 2022-Q3 | 5 | 26.32% |
| BA.1 | × | × | × | Morocco | 2021-Q4 | 11 | 22.45% |
| BA.1.1 | × | × | × | Peru | 2022-Q2 | 12 | 9.92% |
| BA.1 | × | × | × | Mauritius | 2022-Q1 | 5 | 9.80% |
| BA.1.1 | × | × | × | Denmark | 2022-Q2 | 5 | 7.35% |
| AY.127 | × | × |  | Sweden | 2021-Q3 | 8 | 6.61% |
| AY.34.1.1 |  | × |  | Chile | 2021-Q3 | 5 | 5.00% |
| BA.1 | × | × | × | Zambia | 2022-Q1 | 6 | 4.17% |
| BA.2.3 | × |  |  | Philippines | 2022-Q2 | 5 | 4.03% |
| AY.122 | × | × |  | South Africa | 2021-Q3 | 5 | 3.40% |
| BA.5.2.20 | × |  |  | Japan | 2022-Q4 | 5 | 3.36% |
| BA.1 | × | × | × | Greece | 2021-Q4 | 5 | 2.72% |
| BA.5.2 | × | × | × | Singapore | 2022-Q4 | 6 | 2.68% |
| BA.2 | × | × | × | Spain | 2022-Q3 | 7 | 2.27% |
| AY.120 | × | × |  | India | 2021-Q4 | 18 | 2.26% |
| BA.1 | × | × | × | Greece | 2022-Q1 | 11 | 1.86% |
| BA.1.15 | × | × |  | USA | 2022-Q2 | 6 | 1.80% |
| BA.2 | × | × | × | France | 2022-Q3 | 7 | 1.79% |
| BA.1.1 | × | × | × | Brazil | 2022-Q2 | 11 | 1.67% |
| BA.5.2.1 | × | × | × | Vietnam | 2022-Q3 | 5 | 1.49% |
| BA.1.18 | × | × |  | Reunion | 2022-Q1 | 7 | 1.40% |
| BA.1.15.1 | × | × |  | Germany | 2022-Q1 | 13 | 1.39% |
| BA.2 | × | × | × | Malaysia | 2022-Q3 | 5 | 1.38% |
| BC.1 | × |  |  | Japan | 2022-Q2 | 5 | 1.22% |
| BA.1.1 | × | × | × | UK | 2022-Q2 | 10 | 1.22% |
|  |  |  |  |  |  |  |  |

C15*: Both a direct C15 mutation or deletion, or signal peptide mediated cleavage site shift.
