# Supplementary figures and images for: Convergence of immune escape strategies highlights plasticity of SARS-CoV-2 spike
Source: PLoS Pathog. 2023 May 1;19(5):e1011308. doi: 10.1371/journal.ppat.1011308 (PMC10174534; doi:10.1371/journal.ppat.1011308)

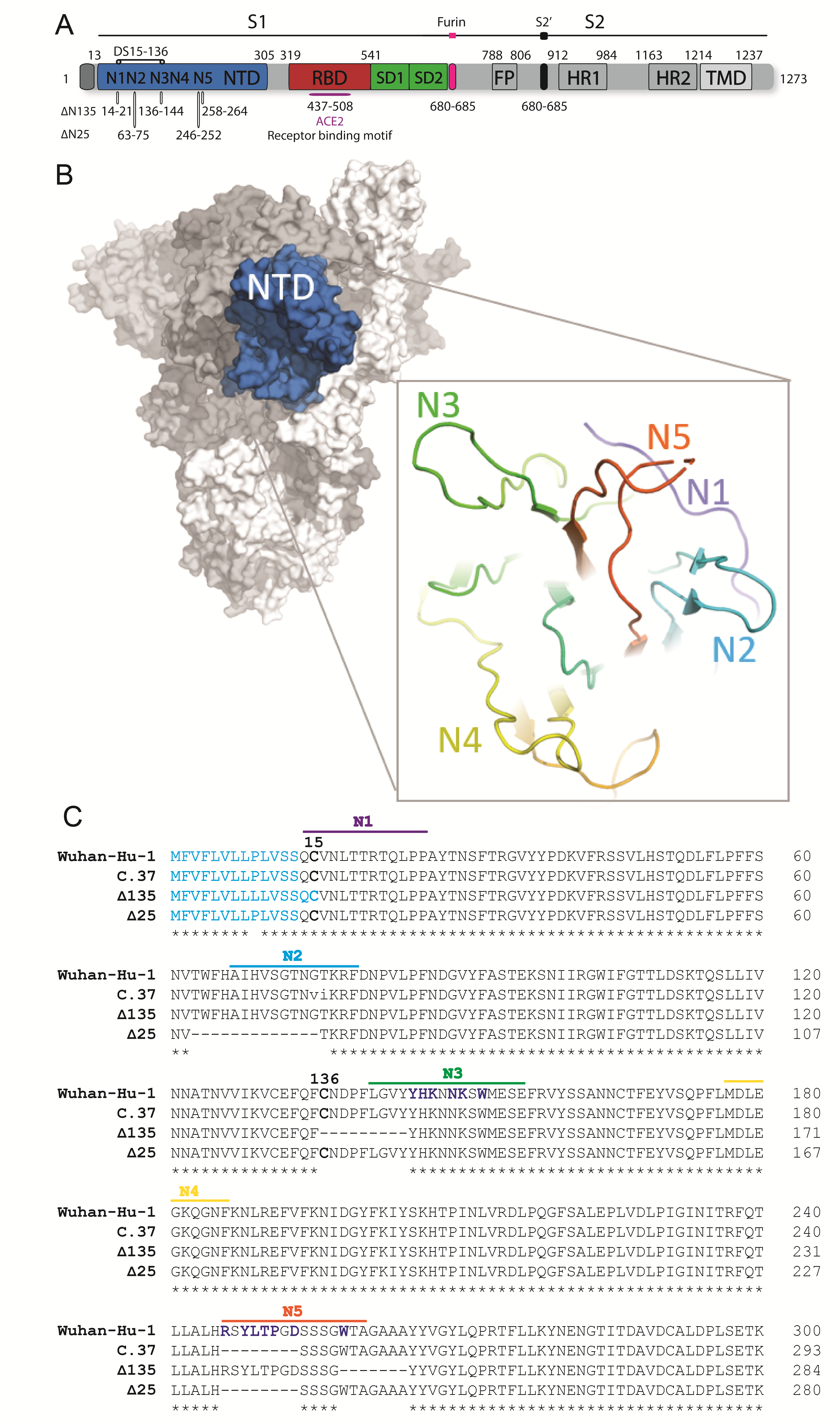

Supplement: S1 Fig — A. Schematic representation of SARS-CoV-2 spikes. SP, signal sequence; DS, disulfide bond; S1/S2, S1/S2 Protease cleavage site; SD1, Subdomain 1; SD2, Subdomain2; S2′, S2′ protease cleavage site; HR1, heptad repeat 1; HR2, heptad repeat 2; TM, transmembrane domain. ACE2 binding site on RBD was highlighted. B. Sideview of a spike with the NTD supersites. C. Sequence alignment of NTD. The sequences have been aligned using ClustalW. Positions of the Supersite N-loops and Cys 15,136 were highlighted above the sequence. Predicted signal peptides and the binding epitopes of neutralization mAbs (4A8, and 2–51) were colored in cyan and dark blue, respectively. (TIF) [file ppat.1011308.s006.tif]

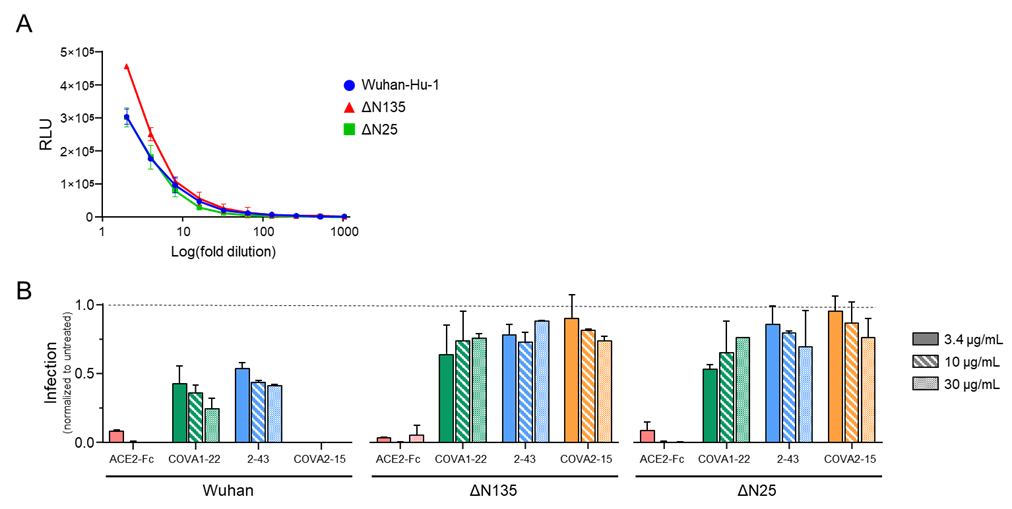

Supplement: S2 Fig — A. Infection of HIV-based lentiviral particles pseudotyped with indicated SARS-CoV-2 Spike on HEK293T_AT cells in a two-fold dilution range. Plotted are relative light units (RLU) based on luciferase expression. B. Neutralization assay with the pseudotyped particles of (A) by ACE2-Fc, COVA1-22, 2–43 and COVA2-15 on HEK293T_AT cells at indicated concentrations. Infection is normalized to the signal obtained for the pseudotyped particle in absence of antibody. (TIF) [file ppat.1011308.s007.tif]

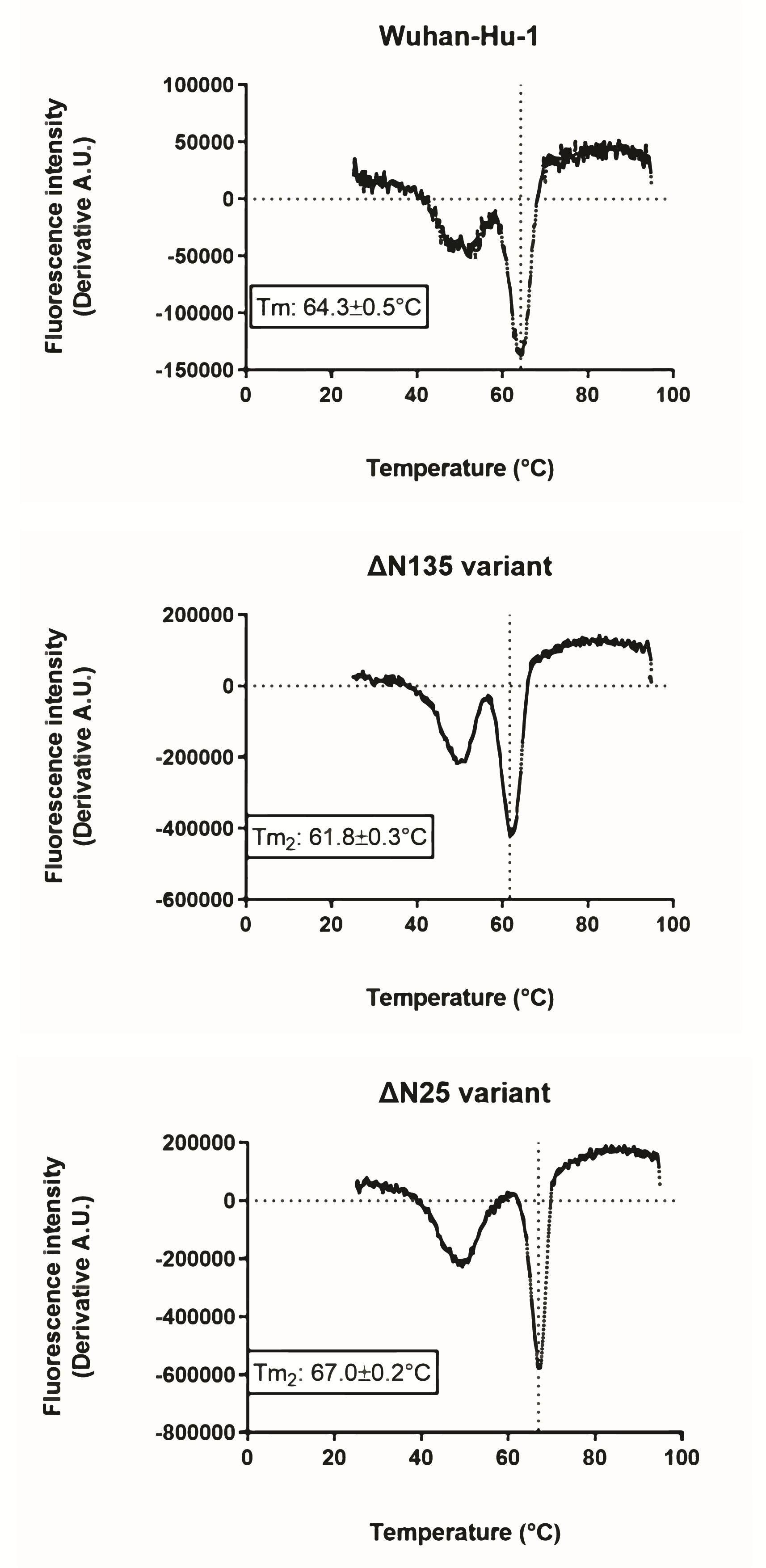

Supplement: S3 Fig — Analysis of melting temperature (Tm) using differential scanning fluorimetry of purified S protein Wuhan-Hu-1 (A), ΔN135 (B) and ΔN25 (C) variants. The first order derivatives are plotted. The experiment was done in triplicate. The Tm is determined as the lowest derivative value representing the Tm50 value. (TIF) [file ppat.1011308.s008.tif]

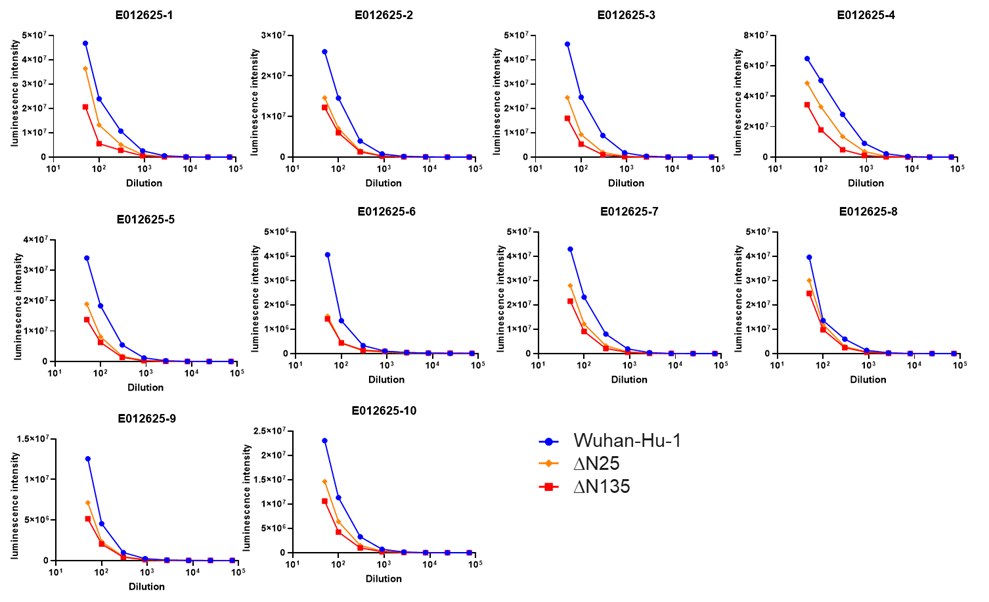

Supplement: S4 Fig — (TIF) [file ppat.1011308.s009.tif]

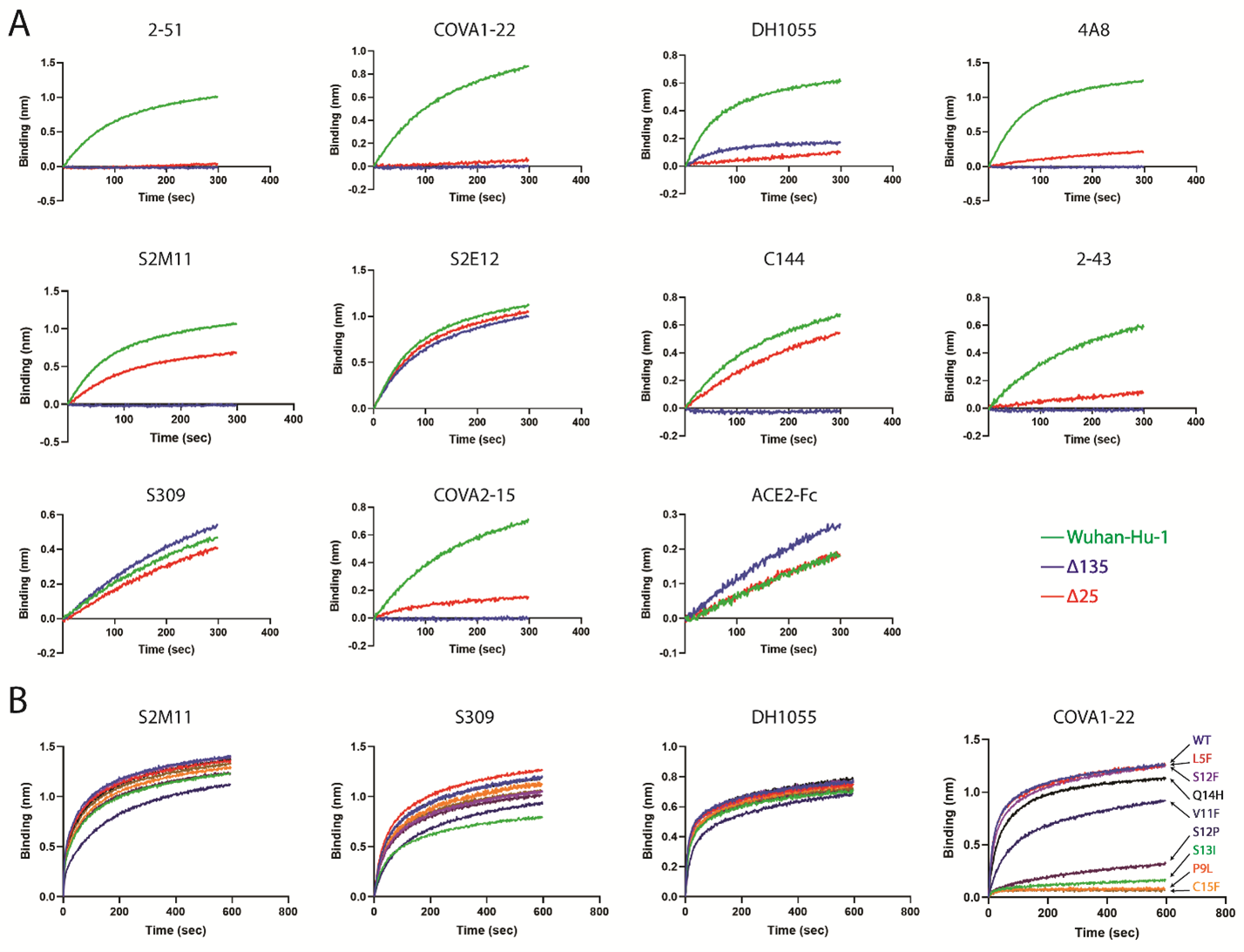

Supplement: S5 Fig — A. Bio-Layer Interferometry curves of the purified Wuhan-Hu-1, ΔN135 and ΔN25 SARS-CoV-2 S proteins binding to ACE2-Fc and a panel of Wuhan-Hu-1 spike binding antibodies. The curves were used to calculate the initial slopes normalized to Wuhan-Hu-1 spike binding plotted in Fig 3. B. Impact of SP mutations on Spike NTD antigenicity. Binding of Mabs COVA1-22, DH1055, S2M11 and S309 to the S trimer with D614G, A892P, A942P and V987P substitutions with the wild type signal peptide (wt SP) and with different mutations in or just after the signal peptide, measured with Biolayer Interferometry (BLI) using Octet, showing the binding curves. The curves are labeled in the right lower panel with the mutations present in the signal peptide or just after the signal peptide. (TIF) [file ppat.1011308.s010.tif]

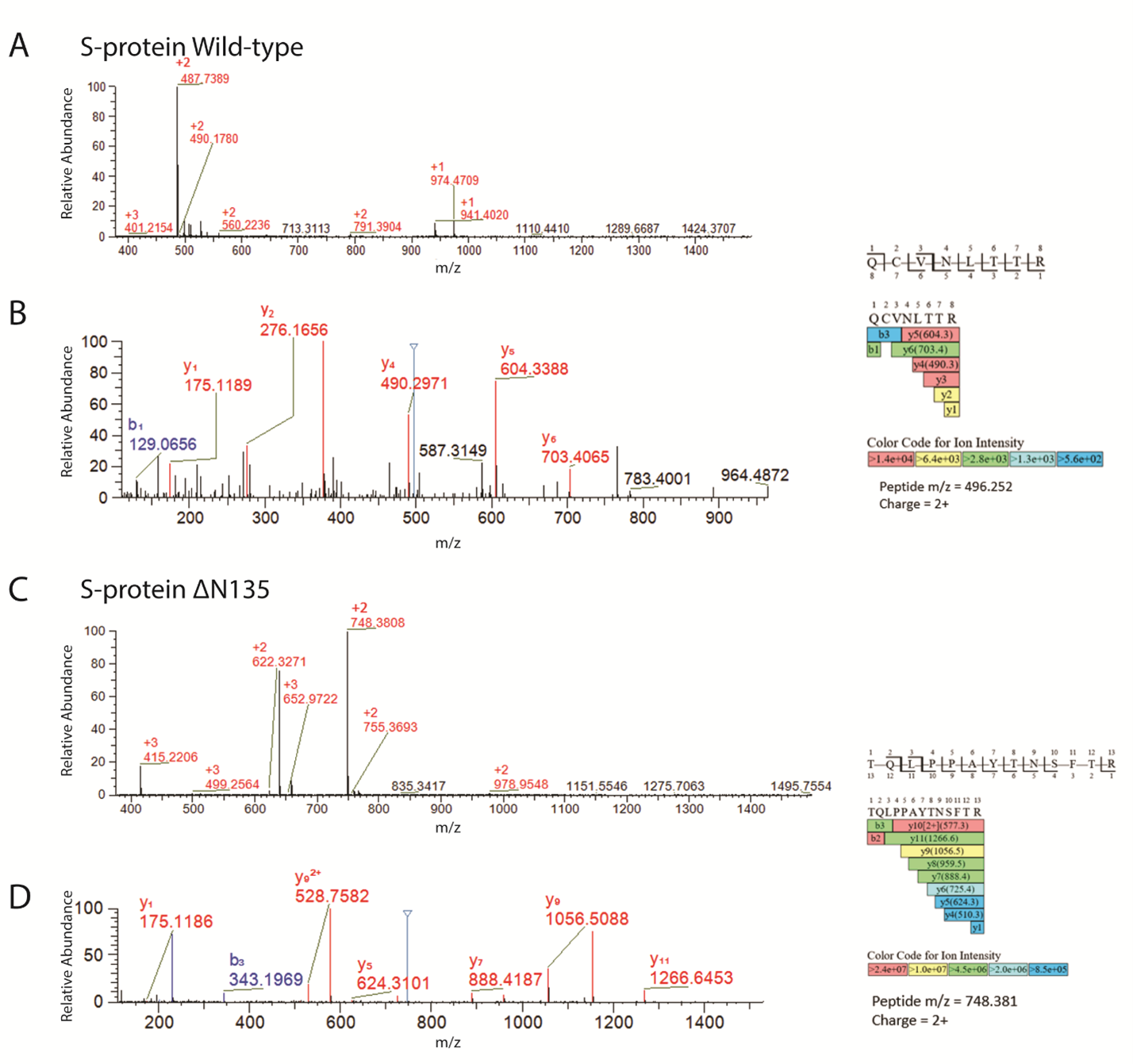

Supplement: S6 Fig — A-B. ESI-MS (A) and MS/MS (B) spectrum of the most N-terminal peptide observed of wild type S-protein treated with trypsin protease. MS/MS analysis shows the MS plot with the most prominent peaks labelled (left) and a list of the identified fragmented peptides (right). C-D. ESI-MS (C) and MS/MS (D) spectrum of the most N-terminal peptide observed of Brazilian variant S-protein treated with trypsin protease. MS/MS analysis shows the MS plot with the most prominent peaks labelled (left) and a list of the identified fragmented peptides (right). (TIF) [file ppat.1011308.s011.tif]

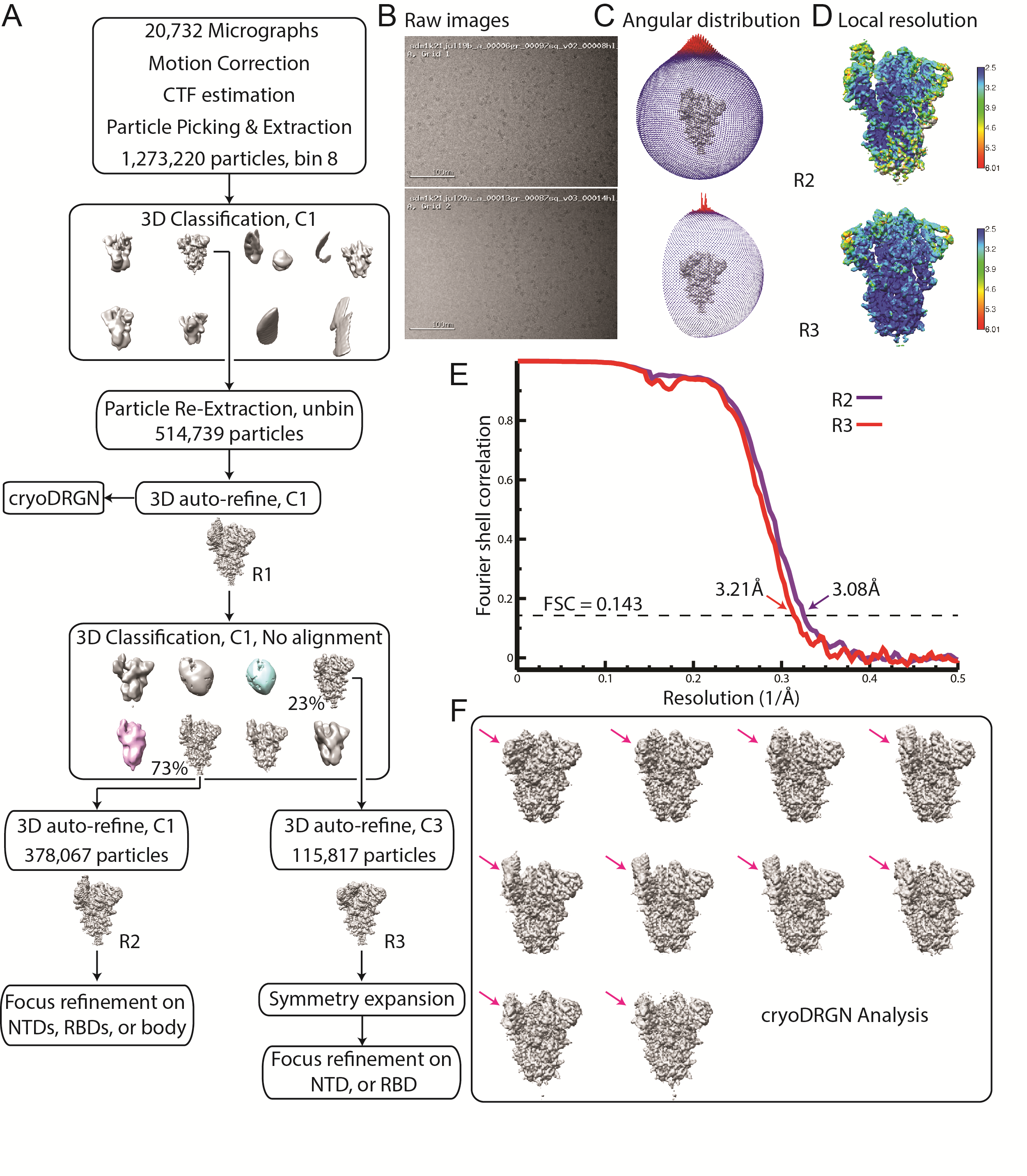

Supplement: S7 Fig — A. Flow chart of the cryo-EM data processing procedure. Details can be found in the Materials and methods. B. A representative cryo-EM micrograph. C. Angular orientation distribution of the particles used in the final reconstruction. The particle distribution is indicated by different color shades. D. Local resolution of the map estimated using the ResMap program and colored as indicated. E. Fourier shell correlation (FSC) curve of the structure with FSC as a function of resolution using Relion output. The resolutions are ~3.08 Å and 3.21 Å at the FSC cutoff of 0.143 for the RBD 1-up and all closed Spikes, respectively. F. cryoDRGN analysis of the ΔN135 spike revealing the mobility of the RBD highlighted in red arrow. (TIF) [file ppat.1011308.s012.tif]

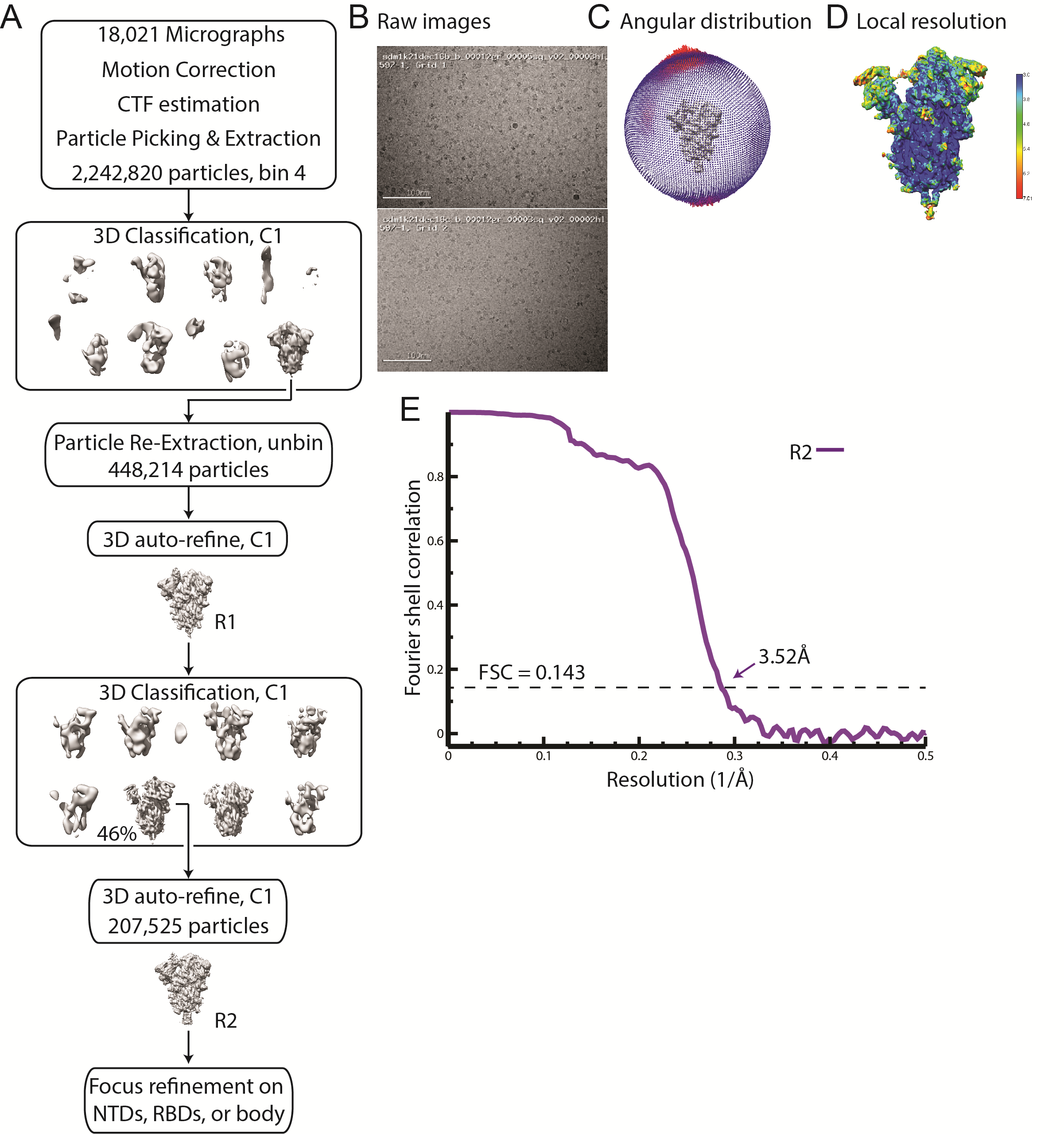

Supplement: S8 Fig — A. Flow chart of the cryo-EM data processing procedure. Details can be found in the Materials and methods. B. A representative cryo-EM micrograph. C. Angular orientation distribution of the particles used in the final reconstruction. The particle distribution is indicated by different color shades. D. Local resolution of the map estimated using the ResMap program and colored as indicated. E. Fourier shell correlation (FSC) curve of the structure with FSC as a function of resolution using Relion output. The resolution is ~3.52 Å at the FSC cutoff of 0.143. (TIF) [file ppat.1011308.s013.tif]

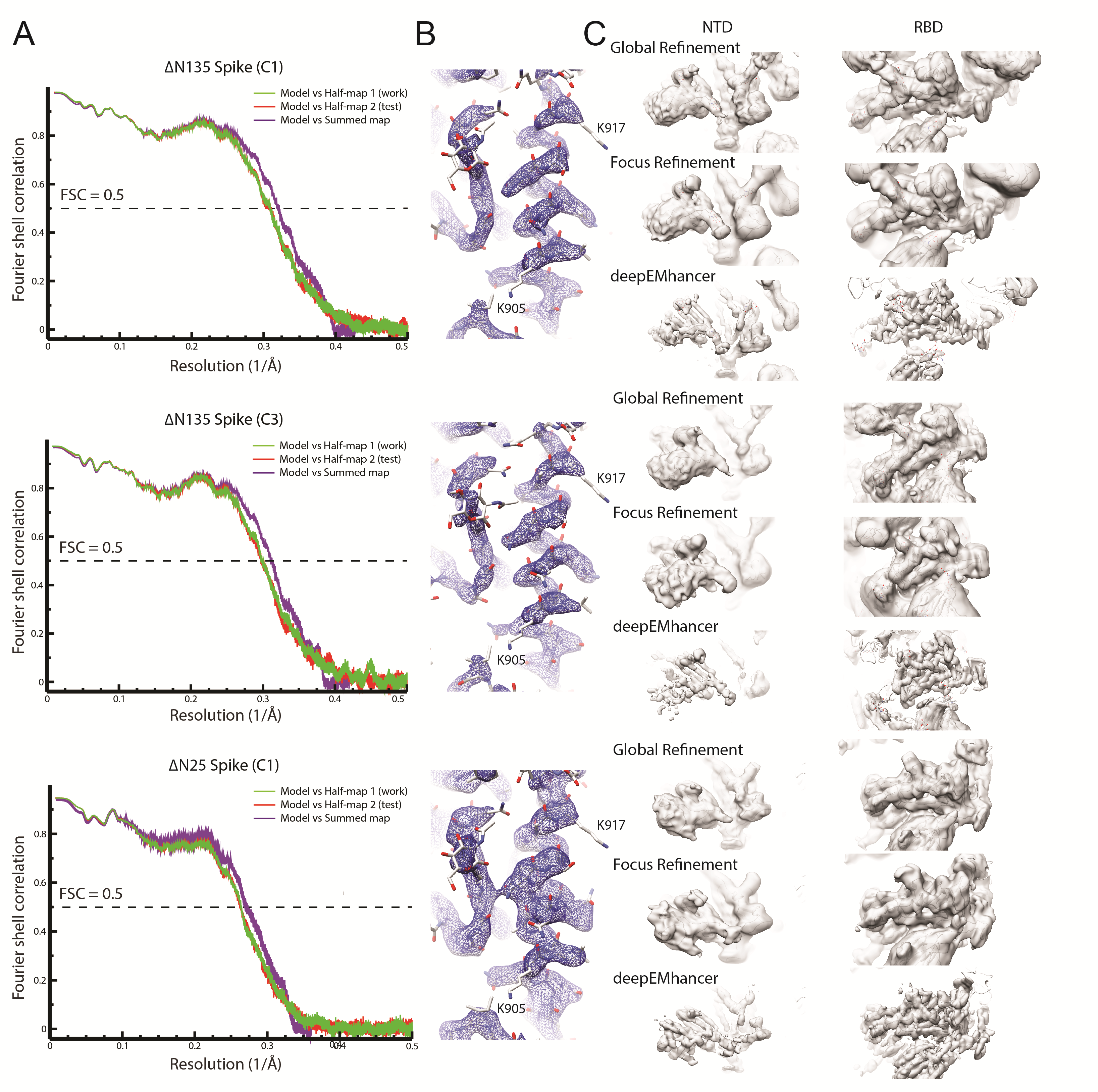

Supplement: S9 Fig — A. Model validation. Comparison of the FSC curves between model and half map 1 (work), model and half map 2 (free), and model and full map are plotted in red, green, and blue, respectively. B. Representative sharpened Cryo-EM density is displayed as mesh at the contour level 15σ. The atomic model with side chains is shown as sticks. C. Representative unsharpened Global refinement, Focus Refinement, and deepEMhancer maps around NTD and RBD were shown as surface at 4.5 σ, respectively. (TIF) [file ppat.1011308.s014.tif]
