# Supplementary material for: Convergence of immune escape strategies highlights plasticity of SARS-CoV-2 spike
Source: PLoS Pathog. 2023 May 1;19(5):e1011308. doi: 10.1371/journal.ppat.1011308 (PMC10174534; doi:10.1371/journal.ppat.1011308)
Supplement: Attachment — Submitted filename: Rebuttal Plos Path.docx [file ppat.1011308.s016.docx]

REVIEWER COMMENTS

**Reviewer #1**

“The authors claim that the spike proteins of the two variants remain fully functional. To support this they only show data from a cell-cell fusion assay with ACE2/TMPRSS2 overexpression and readout 18 hours after transfection. Data from real virus would be interesting to see if replication kinetics are attenuated. Have viruses been isolated or the spike proteins at least been characterized in the context of pseudovirus particles.”

Unfortunately, no viruses with the described deletions have been isolated. In response to the reviewers questions we now generated HIV-based lentiviral particles that were pseudotyped with Wuhan-Hu-1 S, or either of the variant spikes described here (Fig S2). All three pseudotypes were infection competent and could be inhibited by pre-incubation with soluble ACE2-Fc.

*“The quaternary structure of soluble ΔN25 spike was less stable compared to wild type and ΔN135. Is there also an impact on stability of membrane bound spike and potentially replication fitness of the virus.”*

The quaternary structure of soluble ΔN25 spike was indeed less stable compared with the other spikes according to SEC of crude cell culture supernatant but it is difficult to draw strong conclusions from this observation. The spikes are derived from different lineages and therefore it may not be (only) the NTD deletions that determine the spike stability. We have seen strong differences in stability between spikes of different strains that contained stabilizing mutations 986P, 987P and 942P and a furin cleavage site knock out and a C-terminal fibritin foldon trimerization domain, as shown in the figure below.

Moreover, all spikes that were investigated contained very strong stabilizing mutations (prolines at positions 892, 987, and 942 and furin site knock-out mutations) in order to be able to purify and characterize these proteins. The stability measurements and SEC analysis are a good indication that the stabilized spikes we are investigating are well-behaving and comparable to the Wuhan spike. It is an interesting phenomenon that the spike with lower quaternary stability (crude ΔN25 shows more monomer) has highest melting temperature (thermostability). This is something we have observed before with other spike variants and do not completely understand. Somehow such large proteins may somehow lack pliability to form trimers when stabilized. Once the trimers are formed, the stability is very good. We think that this observation is too much besides the message of the manuscript and would result in too much speculation. It would also not be easy to discuss either the lower quaternary stability ΔN25 compared with the higher thermostability. If it needs to be discussed, we think that it should be focused on the higher stability of ΔN25.

Since these variant viruses have not been isolated, we do not know whether replication fitness was affected, but based on before mentioned pseudotyped particles and cell-cell fusion assays we can conclude that they remain functionally active.

“For antigenic characterization binding of soluble spike proteins to monoclonal neutralizing antibodies is analyzed. Also here data from virus particles would be interesting. Is anything known regarding neutralization of ΔN25 and ΔN135 by polyclonal sera.”

We have generated HIV-based lentiviral particles that were pseudotyped with Wuhan-Hu-1 S, or either of the variant spikes described here and show that the neutralization efficiency is greatly impacted (Fig S2)

Minor issues:

*• Line 146-148: Authors speculate that loss of both cysteines in ΔN135 is a compensatory mutation to avoid an unpaired cysteine. However, the next paragraph describes that deletion of one cysteine alone occurs also in other lineages indicating that the loss of Cys136 could also be a coincidence.*

This is a very good point. We have added “this could be” to the text. In many cases only one of the cysteines is lost. However, according to Figure 4B, in many cases eventually both cysteines are lost. For some high-frequency examples like B1.427 and B1.429 a novel cysteine was introduced that is likely pairing with Cys136. We have not investigated all other possibilities of novel pairings or unpaired cysteines but the combined loss of Cys15 and Cys136 is a re-occurring deletion in different lineages.

*• Line 408: Please add reference correctly*

reference corrected

*• Line 450: Please change “Brazilian Spike”*

Changed to ΔN135 spike

*• Figure S4B+C: Please add label to axis*

Original Fig. S4BC was changed to Fig. S6 in the current version with labels added to the axis.

**Reviewer #2**

“How do these mutations affect the binding and neutralizing capacity of different monoclonal antibodies, as well as polyclonal sera following infection and vaccination?”

The effect on the binding of monoclonal antibodies is shown in Figure 2A. For this rebuttal we have generated pseudoviruses and as expected, the neutralizing capacity is mostly determined by mutations in RBD since a pseudovirus assay is known to be especially sensitive for antibodies that are able to block the ACE-2 interaction. We have also included data that show the impact on polyclonal antibody binding in ELISA (Fig. S4).

*“it would be easier to visualize and more informative to plot the prevalence of these deletions in different circulating variants in time”*

We agree with such an approach of data representation. This is what was done for the deletion of the disulfide in Figure 4D. We made an attempt to do this for all (large) deletions in spike but this plot became too complex and was not informative.

other comments:
line 23, remove "all" from " target for all"
line 26, remove " domain" form "NTD domain", as it's redundant
line 28, "we observed" instead of " we observe"
line 29, "in the loss" instead of " in loss"
line 38, replace "sole" with "main"

Thanks for these suggestions. All these items are corrected.

**Reviewer #3**

*“Detailed characterization of recurrent deletions in the SARS-CoV-2 spike, especially in the NTD have been described (DOI: 10.1126/science.abf6950; DOI: 10.15252/embr.202154322). Authors should put their research in the context of prior data and explain the novelty of their findings and how this contributes to the field.”*

We would like to thank the reviewer to bring these papers to our attention. We have included the references in the revised version. The impact of the deletions on the spike function is a very interesting addition in the revised discussion. Our study contributes to these insights and the novelty of our study and additional contribution to the field is the spread of the deletion of the N1 loop and loss of the Cys15-Cys136 disulfide, and the first structural insight how the large deletions impact the overall structure of the NTD. The deletions don’t only shorten the loops but completely repositions the loops.

*“The authors detect substantial reduction in binding of RBD and NTD mAbs against the Spike variants. It would be ideal to assess the impact of these mutations/deletions on neutralization by the panel of antibodies used in this work, using in vitro assays including live viruses or pseudo viruses expressing the corresponding spikes.”*

See previous replies and Figure S2

*“The methods need to be described in detail. As per the current description, it would not be possible to reproduce some of the assays. This is applicable but not limited to the next generation sequencing, protein expression and purification, the BLI, DSF, SEC and fusion assays, which lack a detailed description.”*

Details have been added to the Material and Methods section

*“The specific lineage of the SARS-CoV-2 strain from which the sequences were taken should be indicated.”*

The ΔN25 was derived from the C.37, Lambda variant and the ΔN135 was derived from the B1.1.129 variant as indicated in the first part of the Results section.

*Other comments:*

*Line 56. ‘…obtained from samples OF infected…’
Line 62. ‘in vitro’ should be in italics
Line 329. Please rephrase ‘variants with the cystine bridge absent colored in red’ ◊ ‘variants without the cystine bridge colored in red’*

Thanks for these suggestions. All these items are corrected.
